# Spontaneous subarachnoid hemorrhage: A primer for acute care practitioners

**DOI:** 10.1177/17511437251333269

**Published:** 2025-05-27

**Authors:** Heppner Jonathan, Chwalek Michal, Findlay Max, Brindley Peter George

**Affiliations:** 1 Division of Neurosurgery, Department of Neurosciences, University of Alberta, Edmonton, Canada; 2 Department of Critical Care Medicine, University of Alberta, Edmonton, Canada

**Keywords:** Neuro ICU, subarachnoid hemorrhage, review article, aneurysm, neurosurgery

## Abstract

Subarachnoid hemorrhage (SAH) refers to intracranial bleeding into the cerebrospinal filled space beneath the arachnoid membrane that covers the brain. It is further defined as a spontaneous SAH when not associated with trauma. The commonest single cause is rupture of a saccular (i.e. a small bag-shaped or pouch-shaped) intracranial aneurysm, arising from the larger conducting arteries traveling through the subarachnoid space at the base of the brain. As these are high-pressure and higher-volume arterial hemorrhages, aneurysmal subarachnoid hemorrhages (aSAH) are associated with high early mortality and substantial long-term morbidity. But, as we outline below, prompt and collaborative multidisciplinary care can improve the likelihood and quality of survival. Accordingly, we offer the following primer as a common resource to increase knowledge and collaborative care.

## Introduction

Subarachnoid hemorrhage (SAH) refers to intracranial bleeding into the cerebrospinal filled space beneath the arachnoid membrane that covers the brain. It is further defined as a spontaneous SAH when not associated with trauma. The commonest single cause is rupture of a saccular (i.e. a small bag-shaped or pouch-shaped) intracranial aneurysm, arising from the larger conducting arteries traveling through the subarachnoid space at the base of the brain. As these are high-pressure and higher-volume arterial hemorrhages, aneurysmal subarachnoid hemorrhages (aSAH) are associated with high early mortality and substantial long-term morbidity. But, as we outline below, prompt and collaborative multidisciplinary care can improve the likelihood and quality of survival. Accordingly, we offer the following primer as a common resource to increase knowledge and collaborative care.

## Clinical basics

Ruptured aneurysms account for roughly 10% of all cerebrovascular accidents. Because the subarachnoid space encases the whole brain, the deficits are less localized than after other strokes. Symptoms include sudden-onset severe headache, vomiting, altered level of consciousness, neck pain, numbness, weakness, fever, and seizure (more below). These hemorrhages can be devastating: Up to a quarter of individuals do not even survive to medical care, and two-thirds of those who reach hospital still have unfavorable outcomes.^1,2^

There are other less common types of spontaneous SAHs that resemble aSAH. The most common of these are known as “non aneurysmal,” “perimesencephalic” or “pretrunkal” SAHs. These account for roughly 20% of spontaneous SAHs. In these cases, no aneurysm is found, and the smaller volume hemorrhage is in the subarachnoid space around the upper brainstem (“mesencephalon” refers to the midbrain). Even less common are arteriovenous dural fistulae, where draining veins bridge the subarachnoid space. This is followed by spontaneous intracranial arterial dissections. While rare, these three conditions are always in the differential diagnosis of an aSAH. SAH can also originate from diverse non-aneurysmal sources which include trauma, arteriovenous malformations or fistulae, vasculitis, amyloid angiopathy, and bleeding disorders.^3^ Importantly, the treatment and prognosis of non-arterial SAH is dramatically different, and the outcome usually less injurious.^3^ Accordingly, this review does not cover these alternate etiologies, but does assume they have been ruled out.

The typical aSAH patient is middle aged, and women slightly outnumber men. Risk factors for saccular aneurysm formation and/or rupture include hypertension, include smoking, alcoholism, sympathomimetic drug use (such as cocaine), having had a prior aSAH, and a strong family history of cerebral aneurysms (two or more first degree relatives).^3,4^ There are some heritable conditions associated with intracranial aneurysms, such as Ehlers-Danlos syndrome (vascular subtype) and neurofibromatosis.^3^ These conditions predispose to altered vessel wall integrity, thereby leading to cerebral artery ectasias, and “fusiform” (meaning tapered at both ends) intracranial aneurysms.

Aneurysmal rupture can also lead to blood in the brain parenchyma, ventricles and subdural space in addition to the subarachnoid space. Regardless, these “extended bleeds” are associated with worse prognosis. The most immediate risk of a newly diagnosed aSAH is rebleeding. Following the index rupture, rebleeding within the first 24 h increases mortality up to 40%.^5^ Additional concerns include increased intracranial pressure, spasm of large and small vessels, and impaired CSF drainage causing hydrocephalus. Accordingly, all SAHs should be considered aneurysmal until proven otherwise.

## Unruptured aneurysms

Aneurysms occur in approximately 2%–4% of people worldwide (primarily from autopsy data). The incidence varies among populations; it is reported higher in Japan and Finland, for example. Regardless, the majority are small (<10 mm) and innocuous – hence the general population is not screened. The average risk of rupture of an incidentally discovered (i.e. unruptured) saccular aneurysm is no more than 1%–2% per year, so treatment recommendations are based on lifetime “cumulative risk.” This, in turn, means the overall risk is higher in younger patients. Accordingly, younger patients with higher risk (see immediately below) unruptured aneurysms are likely to be prophylactically repaired, whereas many incidentally discovered aneurysms are more often followed.

Patients are deemed higher rupture risk is they have: (i) a history of rupture from another treated aneurysm, (ii) or larger aneurysmal diameter, (iii) or an irregular shaped aneurysm, or (iv) an aneurysm in a more dangerous location (such as the posterior circulation arteries), or (v) a family history of cerebral aneurysms. Notably, cerebral aneurysms are unrelated to abdominal aneurysms. Regardless, because up to 50% of intracranial aneurysms are discovered incidentally – rather than following an intracranial bleed^6^ – we cannot precisely report the annual rupture rate. While certain events can coincide with aSAH, including exercise, intercourse and cocaine use, nothing is a clear-cut trigger, and patients with unruptured aneurysms are advised to live their lives without activity restrictions.^7^

## Classifying SAHs

aSAHs are classified according to the patient’s signs and symptoms. The original grading system came from Drs. Botterell and Lougheed at the University of Toronto in the 1950s. This helped with standardization, and offered a way to quantify and compare based on presenting “grade.” Soon after, Americans Hunt and Hess introduced a virtually identical system still in use today. This has since been complimented by the World federation of Neurosurgical Societies (WFNS) scoring system. Along with the generic Glasgow Coma Score, (GCS) but specific for aSAH, these grading systems help predict clinical outcome. Currently, the 3 most prevalent scoring/grading systems are the Modified Fisher Scale (mFS), the Hunt and Hess (HH), and the modified World Federation of Neurosurgical Societies (mWFNS). These are compared in Table 1.

**Table 1.** Summary of contemporary SAH grading scales.

Modified WFNS		Modified Fisher			Hunt and Hess		
Grade	Criteria	Grade	Criteria	Symptomatic vasospasm (%)^a^	Grade	Criteria	Survival^b^
1	GCS 15	1	SAH <1 mm, no IVH	24	1	GCS 15	70
2	GCS 14	2	SAH >1 mm, no IVH	33	2	GCS 13–14 with no deficit^a^	60
3	GCS 13	3	SAH <1 mm, with IVH	33	3	GCS 13–14 with deficit^a^	50
4	GCS 7–12	4	SAH >1 mm, with IVH	40	4	GCS 7–12	20
5	GCS <7				5	GCS <7	10

a Deficit may relate to focal weakness, focal paresthesia, or meningismus.

b Survival is described as the average for the corresponding grade during admission for the index SAH.

The mFS system assesses the amount of blood in the subarachnoid space and ventricles and is used to predict vasospasm, or, more specifically, post-SAH delayed onset transient large artery narrowing (see below). The thicker and more diffuse the SAH the higher the risk of vasospasm. Moreover, vasospasm predictability is enhanced when the SAH blood persists over several days, as occasionally a “thick” SAH can decrease over 24 h.^8–10^ The clinical grading systems and radiographic predictions are more sensitive when applied within 6 h of ictus and decrease to 50% sensitivity by 5 days post.^2^

## Clinical presentation

Over half of aSAH patients report a sudden onset “thunderclap” headache, accompanied by nausea, vomiting, and altered consciousness. They can also develop neck stiffness (meningismus) in the hours following due to chemical meningitis.^11–13^ This meningismus is distinct from the acute inflammatory reaction caused by blood in the subarachnoid space at the index event.^14^ For those with a migraine history, the SAH headache may be particularly severe, and is still usually distinct from their usual.^11^ Formerly, small aSAHs that were undiagnosed but followed by major rebleeds were called “sentinel hemorrhages.”^14^ These are now more properly thought of as low volume hemorrhages where the patient remained stable (good grade) and did not seek medical attention. Alternatively, they did present but their condition was not recognized: a serious oversight. After a rupture it is still important to enquire retrospectively about a “sentinel headache,” because this represents time zero for the development of vasospasm/delayed cerebral ischemia.^15,16^ In severe cases, or those affecting the temporal lobe, patients can also seize, which portends worse prognosis and increased mortality.^17^

On examination, patients are frequently hypertensive. Ophthalmoscopy can reveal vitreous hemorrhages (Terson’s Syndrome), which are associated with worse prognosis.^13^ Importantly, absence of the above findings in the presence of a new severe headache does not rule out aSAH: as up to 40% of patients present alert and neurologically intact.^15^ Missed or delayed diagnoses are associated with worsen outcomes, and hence a new “thunderclap” headache should prompt comprehensive evaluation and precautions (more below).

## Diagnosis – general principles

Following history and physical, the best next test is a non-contrast head computed tomography scan (CT). State-of-the art CTs can detect 100% of aSAHs when performed within hours of presentation, and alert diagnosticians pay particular attention to the basal subarachnoid cisterns.^18^ Importantly, sensitivity is highest within 6 h of headache onset and diminishes thereafter. If a CT scan is done because of concern of aneurysm rupture, and is deemed normal, then additional testing follows. Formerly this meant a lumbar puncture (LP), looking for evidence of bleeding in the cerebrospinal fluid (CSF). Increasingly it now means CT-angiography (CTa), in order to interrogate the vessels. By the NICE guidelines, LP is not diagnostic when negative before 12 h after thunderclap headache.^19^

Despite the superiority of modern CTa in SAH workup, LP is still used, especially when CTa is not accessible, or for clinicians who want a second investigation following a CTa that is negative or non-diagnostic. The CSF is examined for xanthochromia,^20,21^ namely CSF discoloration seen with the naked eye after the CSF is centrifuged. Specifically, the practitioner looks for pink or yellow tint in the CSF resulting from blood’s breakdown products (namely bilirubin) and the sample must be protected from light at all times following collection. By 12 h post-aSAH, 100% of patients should have xanthochromia.^19,22^ Considerations that may lead one to forgo LP is s that they can cause pain, which could increase the likelihood of aneurysmal rebleeding. Also, it is not uncommon to get a “traumatic tap” – namely, bleeding into the CSF from needle trauma. This may confuse the diagnosis, and is why, if an LP is performed, it should be done by a skilled practitioner and one able to distinguish a true SAH from a traumatic tap.

As outlined, it takes several hours for intact RBCs to lyse and release hemoglobin into the CSF (which remains in solution after centrifugation). This xanthochromia delay typically passes by the time patients are assessed in the Emergency Department, have a CT scan performed, and have that scan interpreted. It is also helpful to remember that “traumatic taps” are generally associated with only hundreds of red blood cells (RBCs) per µL CSF, in contrast to the thousands after an aSAH. Moreover, to lessen misdiagnosis, the RBC counts between the first and last tubes can be compared. True SAH has a stable or increased RBC count between tubes, whereas a traumatic tap shows a sequential decrease.^23^ Various RBC thresholds have been proposed, but the typical true SAH range is 2400–3600/µL CSF.^24^ Overall, LP has a sensitivity and specificity >90% (97% and 93% respectively) for diagnosing SAH.

## Diagnosis- pinpointing the aneurysmal source

Large volume and diffuse aSAHs often conceal the source of the bleeding, whereas more localized hemorrhages suggest a nearby rupture location. For example, anterior communicating aneurysms (Acomm) result in basal interhemispheric hematomas; middle cerebral aneurysms (MCA) cause sylvian and insular cistern hematomas, and posterior communicating artery aneurysms (Pcomm) leave carotid cistern hematomas. Of note, approximately 20% of patients have multiple cerebral aneurysms.

**Figure 1.**
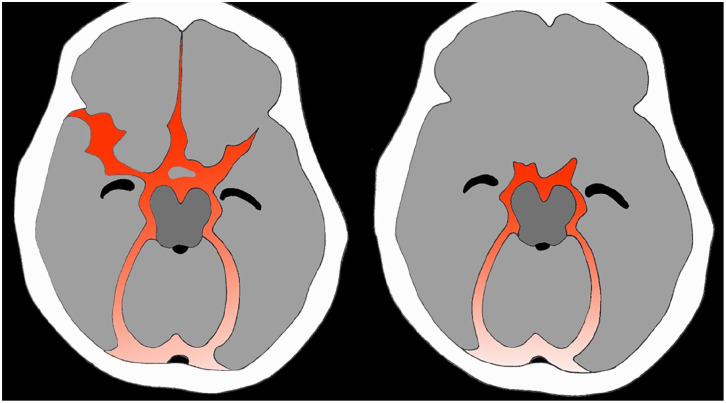
Illustration of characteristic aneurysmal SAH (a) versus perimesencephalic SAH (b).

As mentioned, non-aneurysmal perimesencephalic SAHs have a characteristic pattern of SAH concentrated in the ambient cisterns. When this is seen in alert patients with a normal CTa then no further imaging is required.^25^ These represent 5% of all SAHs. Importantly, they do not confer a morbidity and mortality risk, because they are non-aneurysmal venous bleeds with minimal risk of re-hemorrhage.^26^ Post traumatic SAHs are more often adjacent to the bones of the anterior or middle cranial fossae (Figure 1).^27^

CTa has become the standard of care because it is quick, accessible, and non-invasive. However, small aneurysms (<2 mm) may evade detection.^28^ Digital subtraction angiography (DSA) remains an important addition due to its higher resolution. DSA also allows for treatment to follow diagnosis in one trip, in appropriate cases. In less than 20% of cases, no angiographic source is identified. These so-called “angio-negative SAHs” include cases where the vascular abnormality was obliterated during rupture. Regardless, if the initial imaging (either CTa or DSA) is negative, it is important to repeat the DSA 4–14 days later. This is because up to a ¼ of patients will have an aneurysm detected upon repeat angiography.^25^

## Complications of aSAH and their management

**Figure 2.**
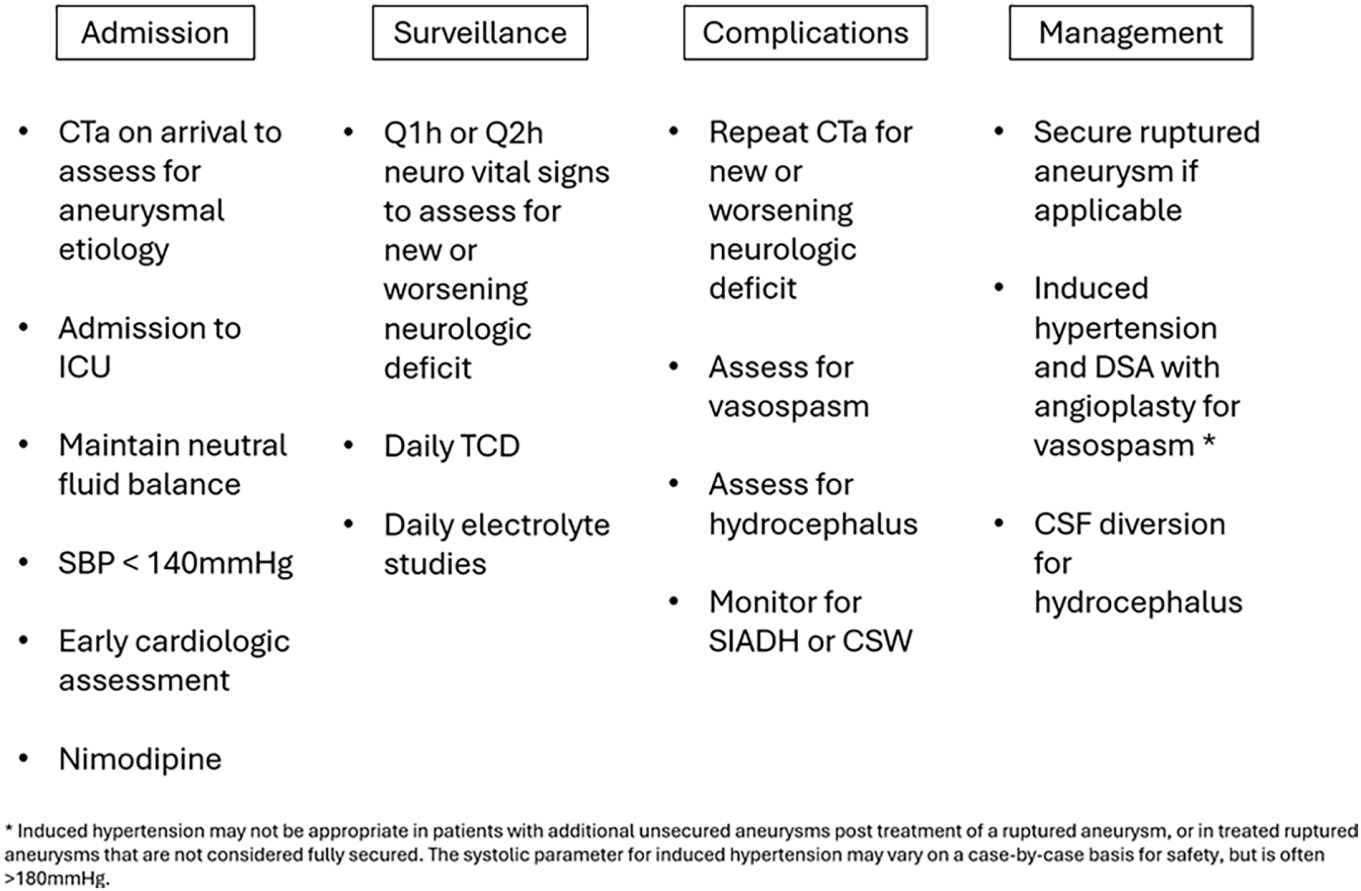
Summary of evidence-based recommendations following a SAH.

Figure 2 summarizes evidence-based management strategies following aSAH rupture. In brief, there are five major risks: re-rupture, vasospasm, raised intracranial pressure (ICP), hydrocephalus and hyponatremia. As with any critical illness, there is stress put upon major organs and all can suffer injury (see below).

Patients appear to do better when admitted to higher volume centers that include experienced staff and collegial multidisciplinary teams.^29^ These teams include emergency department staff, neurovascular surgeons, endovascular specialists, and intensive care practitioners. Prompt referral is therefore recommended, along with an acceptance that early complications – such as hypoxemia, hyperglycemia, fever, and shock – are associated with worse outcome.^30–32^

Acute interventions following aSAH focus on controlling pain and blood pressure. This means intravenous narcotics and antihypertensives in good grade patients, airway control in poor grade patients, and extra ventricular drain (EVD) insertion if there is hydrocephalus. There is insufficient data to recommend routine antifibrinolytic therapy, however it is still commonly used.^33,34^ Neuro exams are commonly performed every 1-to-2 h during the first 2 days, and every 2–4 h during the peak vasospasm period, namely days 3–8.^30^ As outlined, rebleeding is often devastating and usually occurs within 24 h.^35^

**Re-rupture:** The likelihood of rebleed is associated with systolic blood pressure (SBP). Typical blood pressure management is to keep the SBP <140 mmHg (despite some studies suggesting no increased rebleed risk until the systolic exceeds 160 mmHg).^36^ Brain perfusion more closely correlates with mean arterial pressure (MAP)and typically a target of cerebral perfusion pressure (CPP) >65 mmHg (calculated by MAP-ICP) is chosen.^37^ Venous thromboembolic prophylaxis begins with pneumatic mechanical methods, and transitions to chemical prophylaxis once the aneurysm is secured.

An aneurysmal rebleed can mimic a generalized seizure. As such, a repeat CT scan should be performed with any neurologic deterioration. Seizures should be treated but seizure prophylaxis is less clear.^38^ Seizures are more likely if there is thick subarachnoid clot, intracerebral extension, delayed infarction, middle cerebral artery proximity, high-grade SAH (Hunt & Hess >3 or Fisher III/IV), cortical infarction, or hydrocephalus.^30,39,40^ Phenytoin is being used less because of worse cognitive outcomes and, accordingly, levetiracetam is being used more.^39–41^

**Figure 3.**
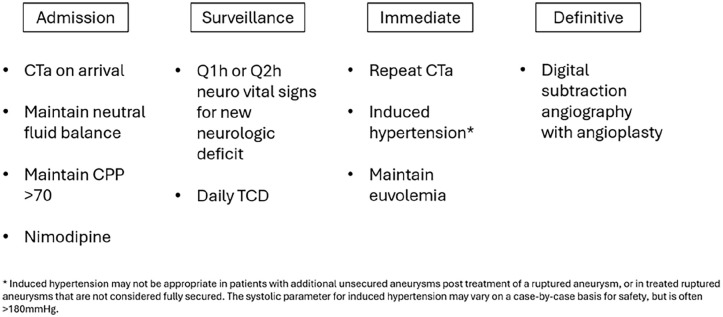
Summary of evidence-based recommendations for managing vasospasm following aSAH.

**Vasospasm:** Cerebral vasospasm is a narrowing of the cerebral arteries that can be severe. It is ultimately reversible, but, if unchecked it can substantially increase morbidity and mortality^16,42^ and cause irreversible delayed cerebral ischemia (DCI). Vasospasm begins several days after SAH, peaks in severity approximately 7 days later, and then resolves by about 14 days. Figure 3 summarizes vasospasm management. The trigger is not conclusively known but likely relates to blood breakdown products eliciting an inflammatory response to the outside of intracranial arterial walls.^16^ The risk of vasospasm/DCI depends mainly on the thickness of blood clots in the subarachnoid space and ventricles (a high Fisher grade, as discussed above).^9^ Progression to DCI depends on the degree of arterial narrowing (mild, moderate, or severe) and its distribution (focal or diffuse).

Vasospasm remains one of the important determinants of outcome following aSAH even though advances in diagnosis and treatment have reduced resultant morbidity and mortality. The calcium channel antagonist nimodipine is the only proven agent for vasospasm prophylaxis.^30^ As such it is standard following aSAH, although its impact on patient outcome is modest, and it does little once vasospasm is established.^30,43–45^ Despite its vasodilatory properties, nimodipine’s impact on large vessel spasm is uncertain.^45^ The recommended dose is 60 mg administered every 4 h orally (or via nasogastric tube).^30^ If significant fluctuations in blood pressure occur with standard dosing then it is common to change to 30 mg every 2 h. Regarding fluid management, the goal is to maintain euvolemia. Previously, hypervolemia was recommended; however, “prophylactic hypervolemia” is now understood to be harmful and is hence contraindicated.^42,46–48^ In cases of excessive diuresis or natriuresis, fludrocortisone or hydrocortisone may help, though evidence is limited.^49,50^

Regular bedside clinical assessment is key for detecting vasospasm. It can be supplemented by serial transcranial Doppler^51^ and, when appropriate, additional angiography. Development of an arm drift or dysphasia can signal internal carotid artery (ICA) and/or MCA vasospasm, while somnolence and abulia indicate possible anterior cerebral artery (ACA) spasm. Transcranial Doppler velocities that are trending up over several days to over 150 cm/s are concerning, such that even asymptomatic patients will may receive a CTa.^51,52^ TCDs have several important limitations, and as such even trends in velocity elevation are insufficient to definitively diagnose vasospasm in isolation. TCD accuracy is limited by technician experience and inter-technician variation. Accordingly, TCDs are most useful when performed serially by the same experienced technician. TCDs are also limited by normal variations in skull thickness and structure which impairs adequate interrogation of one or more major vessels (namely, “inability to get a good window”). Additionally, TCDs are most reliable in large diameter vessels such as the internal carotid and middle cerebral arteries, but less reliable in smaller vessels. Because of these limitations, TCDs are best regarded as a supplemental way to monitor for vasospasm. These limitations mean that some guidelines (including the NICE guidelines) recommend against utilization of TCDs in vasospasm monitoring,^19^ thereby highlighting the controversy around this imaging technique.

For patients who are poor grade and/or sedated, where a neurological exam is difficult, we routinely undertake at least two CTa’s looking for vasospasm during the vasospastic interval: days 5–12 post SAH. Importantly, not all radiographic vasospasm leads to clinical changes, and not all patients with clinical symptoms (consistent with vasospasm) have spasm on CTa. For this reason, CTa is important but not definitive in the workup of patients for whom vasospasm is suspected (except where stated above). I Instead, the approach is to induce hypertension and perform DSA.

Symptomatic vasospasm (in patients who can be examined) should be treated immediately with induced hypertension. Targets are individualized but are usually set to the lowest systolic blood pressure required to reverse the neurologic deficit. Induced hypertension alone may be sufficient, and noradrenaline or phenylephrine are the preferred vasoactives. Milrinone – an inodilator – is also used to combat vasoconstriction or if there is myocardial dysfunction.^53–56^ If deficits are not reversed within an hour after a target blood pressure (i.e. 200 mmHg) is reached, then endovascular therapy (balloon angioplasty) is indicated to prevent cerebral infarction (see Table below). There is insufficient evidence at time of writing to dictate the timing at which endovascular therapy after vasospasm, though earlier intervention is presumed to be better from the “time is brain” perspective – namely, that a longer ischemic time could mean more parenchymal damage. As such, in the absence of strict guidelines, we suggest that endovascular intervention should be undertaken urgently regardless of time of day and following liaison with the neurointerventional staff when required.

For patients who cannot be properly assessed by clinical examination, moderate or severe angiographic vasospasm should trigger urgent balloon angioplasty.^57^ If a patient fails to improve with hypertension and/or balloon angioplasty then it is likely that they have an established cerebral infarct. The caveat is that other systemic issues can confound (i.e. hypercarbia, electrolyte or glucose disturbance, sepsis). Following aSAH there is a tendency to assign any type of delayed onset worsening to “vasospasm” when it could be another cause.

**Raised intracranial pressure:** SAH can lead to hydrocephalus and hematomas which can elevate ICP. This was discussed above in regards to acute management, and will now be covered in regards to chronic management. In short, large bleeds can impair CSF drainage. AComm aneurysms can cause large frontal hematomas, and MCA aneurysms can cause temporal lobe or frontotemporal hematomas. Surgical evacuation may be required, including large craniectomies for select patients either bifrontal (for Acomm aneurysm) or frontotemporal (for MCA aneurysms). This is because localized swelling can cause a marked ICP rise. These major operations constitute a desperate life-saving measure in the face of intractable intracranial hypertension, rather than a routine intervention. Poor grade patients with more severe SAHs can also suffer diffuse brain swelling, which also progressively worsens intracranial hypertension. Elevating the head of the bed, and ensuring that the neck is free from constriction, can temporize the patient prior to definitive management. Patients may need to be sedated and sometimes paralyzed to control ICP, with the caveat that this limits the neurological exam to pupil checks only.

Hyperosmolar treatment is common, whether with hypertonic saline (3%) or intermittent mannitol, or both. Mannitol is an intermittent bolus whereas hypertonic saline is either an intermittent bolus or continuous infusion. There is no clear evidence of one osmotherapy being superior to another, but all require close monitoring of serum sodium concentrations (see below) and renal function.

**Systemic manifestations:** Hyponatremia is common following SAH, and likely results from hypothalamic injury.^58^ Patients may present with either the syndrome of inappropriate antidiuretic hormone secretion (SIADH) or cerebral salt-wasting syndrome (CSW). Both conditions present with low serum sodium and inappropriately high urine sodium. The difference is that patients with SIADH are euvolemic, while those with cerebral salt-wasting are hypovolemic.^58,59^ The concern with hypertonic sodium repletion is the possibility of profoundly disability from central pontine myelinolysis. This is associated with rapid serum sodium rise, and is more common in patients with chronic hyponatremia, alcoholism, and liver disease. Typically, hypertonic saline is stopped once the serum sodium is over 135 or if it rises by more than 2 mMol/L per hour. Fludrocortisone can also be used in cerebral salt-wasting to mitigate the over-diuresis that distinguishes this syndrome from SIADH.^50,59^

Cardiopulmonary complications affect over a third of SAH patients. These include ventricular dysfunction, arrhythmias, pulmonary edema, acute respiratory distress syndrome (ARDS), and even sudden cardiac death.^13,60–62^ Electrocardiographic changes are heterogenous: including QT prolongations, T wave abnormalities, ST elevations and depressions. Increased troponin levels are associated with increased cardiopulmonary complications; and Takotsubo cardiomyopathy is characterized by apical ballooning of the left ventricle – and presumed to be from sympathetic excess.^60,61^ The high incidence of cardiopulmonary complications is another reason for vigilance, along with a low threshold for echocardiography, especially in unexplained shock.^61,63,64^ Lung management mirrors usual ICU care, except that permissive hypercapnea and high PEEP might increase ICP.^65^

Hyperthermia is common whether from cerebral inflammation, hypothalamic damage or secondary infection, especially in high grade bleeds. It is also associated with worse outcome.^66,67^ Therefore, we closely monitor body temperature (typically via an esophageal probe) and have a low threshold for antibiotics, antipyretics (usually paracetamol) and cooling blankets.

**Hydrocephalus:** Approximately one-third of aSAH patients develop acute hydrocephalus. This is due to blockage of subarachnoid pathways by hematoma and erythrocytes (external hydrocephalus) or by ventricular hematomas (internal hydrocephalus).^68,69^ Hydrocephalus is more likely following larger volume SAHs, intraventricular hematomas, poorer clinical grade, and Acomm aneurysms.^69,70^ Research is underway regarding prophylactic CSF removal via lumbar drainage,^71,72^ but, to date, there is insufficient consensus to recommend this.

EVDs are indicated for all SAH patients with ventricular dilatation, especially if high grade. If the plan is to manage the aneurysm endovascularly (see below) then the EVD should be inserted in the emergency room, ICU, or operating theater (depending on urgency and local practice requirements). Alternatively, the EVD can be inserted simultaneously intraoperatively when there is an open surgery for decompression of an associated hematoma. Intraventricular fibrinolysis, using recombinant tissue plasminogen activator (rt-PA), has been shown to rapidly clear large aneurysmal IVHs and improve ICP and maintain ventricular catheter patency.^73^ The typical dose is 4 mg in 4 mL of saline, administered via the EVD directly, and with the EVD closed and opened over 2 h.

Patients at high vasospasm risk require a longer period of drainage, often 10–14 days. In contrast, low risk patients (those with less clot burden) can have their EVD removed sooner. There are three common weaning strategies (without evidence of superiority^70,74^). The first approach involves a gradual raising of the height of the drain/gradual increase in resistance over several days. The second approach is rapid whereby the EVD is abruptly clamped and only reopened/drained in the event of clinical deterioration. The third approach is in between, wherein the EVD height is gradually raised until the daily drainage is <150 mL/24 h (the daily production of CSF is approximately 500 mL) with the drain set at 20 cm of H_2_O (normal ICP is less than 20). At this point the drain is clamped (at 20 cmH_2_0) and only reopened for 15 min (still at a height of 20 cm H_2_O) and only in the event of unequivocal neurological decline: and not for headache alone, or for an isolated ICP measurement. A CT scan is then done the following day to rule out hydrocephalus, and if absent then the EVD is removed. This third approach is s local practice at our institution in Canada, and is based on decades of trial and error of the other two approaches. While the superiority of these approaches has not been scientifically proven,^75^ we have found it to be a safe and reliable approach. .

Failure of EVD wean occurs in approximately 20% of SAH patients, thereby necessitating a long-term CSF diversion.^69^ The need for shunt is not affected by whether the aneurysm was treated with surgical clipping or endovascular coiling.^76,77^ Regardless, this procedure typically involves a subcutaneous catheter placed from brain to abdomen, otherwise known as a ventriculoperitoneal shunt, or VP shunt.^74,77,78^ The peritoneal space is generally easy to access and has more than adequate absorptive capacity.

## Aneurysm repair

The options for aneurysm repair are either endovascular coiling or microsurgical clipping. The choice is guided by patient condition and age, along with aneurysm location, size, and width of neck. Currently, coiling and clipping appear equally effective.^79^ Endovascular coiling is favored for poor grade and older patients, and small neck aneurysms. There is still an important role for aneurysm clipping, however, especially in younger patients and for large and wide neck aneurysms, which are harder to obliterate with coils. Surgery is also required for patients with large intracerebral hematomas requiring evacuation due to mass effect (discussed above).^67^ It is beyond our scope to outline the various endovascular interventions and surgical approaches, but excellent reviews exist.^80,81^ The timing of aneurysm repair following rupture has been extensively discussed in the literature. In brief, it is typical to repair the aneurysm within 24 h, as per the 2023 American Heart and Stroke Association guidelines^30^However, generally, repair need not be emergent – as the re-rupture rate is the highest within the first hour following index rupture – unless there is an compressive hematoma that requires evacuation,

## In closing: Post ICU care

This review article focuses on acute care fundamentals following aSAH. This is in no way to minimize the profound importance of post ICU care, neuro rehabilitation, and the need to address the pastoral needs of patients and loved ones. aSAH can be devastating, but can also be an excellent example of multidisciplinary care throughout the patient and family’s complex course.
